# Supplementary material for: An open-source framework for large-scale, flexible evaluation of biomedical text mining systems
Source: J Biomed Discov Collab. 2008 Jan 29;3:1. doi: 10.1186/1747-5333-3-1 (PMC2276192; doi:10.1186/1747-5333-3-1)
Supplement: Additional file 1 — Evaluation platform source code. The additional file contains the software evaluation framework discussed in this paper. The most current release can be downloaded from SourceForge.net [1]. See the accompanying README file for instructions on installation and use. Also included in the distribution is a collection of UIMA wrappers for some commonly used BioNLP tools and annotated corpora. [file 1747-5333-3-1-S1.zip › bionlp_uima_repository-1.0.1/docs/api/edu/uchsc/ccp/util/external/stanfordparser/StanfordParser_Util.html]

StanfordParser\_Util (Center for Computational Pharmacology Util/UIMA API)


|  |  |  |
| --- | --- | --- |
| **Overview** \| **Package** \| **Class** \| **Tree** \| **Deprecated** \| **Index** \| **Help** | |  |
| **PREV CLASS**   **NEXT CLASS** | **FRAMES**    **NO FRAMES**     **All Classes** |  |
| SUMMARY: NESTED \| FIELD \| CONSTR \| METHOD | DETAIL: FIELD \| CONSTR \| METHOD |  |


---


## edu.uchsc.ccp.util.external.stanfordparser Class StanfordParser\_Util

```
java.lang.Object
  edu.uchsc.ccp.util.external.stanfordparser.StanfordParser_Util
```

---

``` public class StanfordParser_Util extends Object ```

This class converts the output of the StanfordParser into a distinct text annotation objects. The main purpose for this conversion is to
incorporate the StanfordParser into the UIMA framework.   
  
Obtain the StanfordParser here: http://nlp.stanford.edu/downloads/lex-parser.shtml
The output of this class is an ArrayList of TextAnnotations. Each TextAnnotation will have a corresponding ClassMention associated with
it. The ClassMention will either be a token mention with partOfSpeech slot, a phrase with phraseType slot, or a clause mention with
clauseType slot.
NOTE: Phrase/Clause Levels are incorrect.

**Author:**
:   William A. Baumgartner, Jr.

---

**Constructor Summary**

`StanfordParser_Util(String serializedGrammarFile)`
  
          Construct a new StanfordParserUtil.

`StanfordParser_Util(String serializedGrammarFile,
boolean attachTypedDependencies)`

`StanfordParser_Util(String serializedGrammarFile,
String outputFormats)`
  
          Construct a new StanfordParserUtil while selecting the output tree display format.

`StanfordParser_Util(String serializedGrammarFile,
String outputFormats,
boolean attachTypedDependencies)`


| **Method Summary** | |
| --- | --- |
| `static void` | `main(String[] args)` |
| `List<TextAnnotation>` | `parse(String sentence)`             Call this method to parse an inputted string of text. |
| `List<TextAnnotation>` | `parsePartiallyTaggedText(String partiallyTaggedText, String untaggedSentence)`             Not currently functional |

**Methods inherited from class java.lang.Object**

`clone, equals, finalize, getClass, hashCode, notify, notifyAll, toString, wait, wait, wait`

**Constructor Detail**

### StanfordParser\_Util

```
public StanfordParser_Util(String serializedGrammarFile)
```

:   Construct a new StanfordParserUtil.

    **Parameters:**: `serializedGrammarFile` - - Input file location. To use the PCFG parser, this file should be englishPCFG.ser.gz. To use the lexicalized parser, this file should be englishFactored.ser.gz

---


### StanfordParser\_Util

```
public StanfordParser_Util(String serializedGrammarFile,
                           boolean attachTypedDependencies)
```

---


### StanfordParser\_Util

```
public StanfordParser_Util(String serializedGrammarFile,
                           String outputFormats)
```

:   Construct a new StanfordParserUtil while selecting the output tree display format.

    **Parameters:**: `serializedGrammarFile` - Input file location. To use the PCFG parser, this file should be englishPCFG.ser.gz. To use the lexicalized parser, this file should be englishFactored.ser.gz: `outputFormats` - a comma-delimited list of StanfordParser output formats options: penn,wordsAndTags,typedDependenciesCollapsed,oneline,words,dependencies,typedDependencies,latexTree,collocations

---


### StanfordParser\_Util

```
public StanfordParser_Util(String serializedGrammarFile,
                           String outputFormats,
                           boolean attachTypedDependencies)
```


**Method Detail**

### parse

```
public List<TextAnnotation> parse(String sentence)
```

:   Call this method to parse an inputted string of text.

    :   **Parameters:**: `sentence` - text to parse

---


### parsePartiallyTaggedText

```
public List<TextAnnotation> parsePartiallyTaggedText(String partiallyTaggedText,
                                                     String untaggedSentence)
```

:   Not currently functional

    :   **Parameters:**: `partiallyTaggedText` -: `untaggedSentence` - **Returns:**

---


### main

```
public static void main(String[] args)
```

:   **Parameters:**: `args` - args[0] = serialized grammar file args[1] = text to parse args[2] = [OPTIONAL] output tree display format


---


|  |  |  |
| --- | --- | --- |
| **Overview** \| **Package** \| **Class** \| **Tree** \| **Deprecated** \| **Index** \| **Help** | |  |
| **PREV CLASS**   **NEXT CLASS** | **FRAMES**    **NO FRAMES**     **All Classes** |  |
| SUMMARY: NESTED \| FIELD \| CONSTR \| METHOD | DETAIL: FIELD \| CONSTR \| METHOD |  |


---
